# CLL cell-derived soluble factors do not influence the functionality of normal B cells

**DOI:** 10.3389/fimmu.2026.1794418

**Published:** 2026-05-15

**Authors:** Michelle Maria Elbert, Bettina Budeus, Maria Dampmann, Nele Kahlert, Artur Kibler, Gero Knittel, Hans Christian Reinhardt, Ralf Küppers

**Affiliations:** 1 Institute of Cell Biology (Cancer Research), University Medicine Essen, Essen, Germany; 2 Department of Hematology and Stem Cell Transplantation, University Medicine Essen, Essen, Germany; 3 Department of Medical Oncology, University Hospital Essen, West German Cancer Center, Essen, Germany; 4 Department of Immunodynamics, Institute for Experimental Immunology and Imaging, University Medicine Essen, Essen, Germany; 5 German Cancer Consortium (DKTK), site Essen, Essen, Germany

**Keywords:** activation, CLL, functionality, leukemia, normal residual B cells, proliferation

## Abstract

**Introduction:**

Humoral immunodeficiency is frequently observed in patients affected by chronic lymphocytic leukemia (CLL). The reasons for the compromised B-cell responses are only partly understood. We hypothesized that the normal residual B cells in CLL patients are negatively influenced in their functionality by soluble factors produced by CLL cells.

**Methods:**

We performed functional B-cell assays to analyze the influence of serum from CLL patients or conditioned medium from CLL cells cultivated with stromal cells on B cells from healthy donors. Serum from healthy individuals or conditioned medium from normal B cells was used as controls, respectively. We stimulated the B cells in a T-cell-dependent fashion and incubated them in the respective serum or conditioned medium for 5 days. We measured plasma cell differentiation, proliferation, cell death, and B-cell activation, the latter using the markers CD80, CD86, and CD25.

**Results:**

We did not detect significant differences supporting the hypothesis that CLL-derived soluble factors negatively influence the proliferation, activation, or cell death levels of normal B cells from healthy individuals in *in vitro* coculture.

**Discussion:**

Our analysis suggests no major direct inhibitory effects of CLL-derived soluble factors on normal mature B cells. Thus, it is likely that other factors lead to the known B-cell dysfunction in CLL.

## 1 Introduction

Chronic lymphocytic leukemia (CLL) is a low-grade B-cell non-Hodgkin lymphoma (B-NHL) and the most common leukemia among elderly individuals in the Western world. The age at first diagnosis is, on average, 70 years (1). CLL is characterized by an accumulation of CD5^+^ malignant B cells in the bone marrow, blood, and lymphoid organs of the patients (2). The immunoglobulin heavy chain variable (IGHV) gene mutation status of the CLL clone has clinical relevance, as unmutated IGHV genes are known to correlate with a worse prognosis (3–5). CLL is considered a slow-progressing but still incurable lymphoid malignancy. At first diagnosis, often with inactive, asymptomatic disease, a watch-and-wait strategy is employed. If patients develop severe symptoms, such as anemia, thrombocytopenia, extreme fatigue, or uncontrolled weight loss, treatment is initiated. Treatment options include targeted drugs such as Bruton’s tyrosine kinase (BTK) and BCL2 inhibitors, used as monotherapy or in combination with monoclonal anti-CD20 antibodies (6).

One hallmark of CLL is a progressive immunodeficiency. This immunodeficiency can develop independently of treatment status and leukemic burden, but it can be exacerbated by them. It involves the B-cell arm of the immune system and leads to recurrent infections, often of the urinary or respiratory tract, which are among the most common causes of death in CLL patients (7–9). Part of the humoral immune deficiency is hypogammaglobulinemia, which typically worsens over the course of the disease and can predict early death and time to first treatment (10–12). Furthermore, the response to vaccination in CLL patients is limited, and negative effects on plasma cells can be detected early in monoclonal B-cell lymphocytosis, a CLL precursor lesion, and early-stage CLL (13–15).

CLL cells secrete factors that influence surrounding blood cells, such as neutrophils, T cells, and natural killer cells (8, 16). CLL cells secrete CCL3, CCL4, IL-8, PDGF, and VEGF, that were identified in coculture systems with nurse-like cells or mesenchymal stromal cells, and produce extracellular vesicle (17–23). CLL cells also lead to differential cytokine secretion by surrounding cells, for example, increased TGF-β1 and CCL2 secretion by stromal cells in coculture with CLL cells (24–26).

It is known that CLL cells depend on signals of their tumor microenvironment, especially from stromal cells in the bone marrow and lymph nodes, for their survival. Several options exist to mimic this stroma *in vitro*. In addition to human bone marrow cells, nurse-like cells that arise from monocytes in the presence of CLL, or stromal cell lines like THP-1 or HS-5, can be used to prolong CLL cell survival (26–29). HS-5 is a stromal cell line with a transcriptome similar to that of bone marrow mesenchymal stromal cells and has similar effects on surrounding immune cells (30), making it a frequently used model for recapitulating stromal cells in CLL coculture systems.

We performed functional *in vitro* B-cell assays using B cells from healthy individuals, which were incubated with serum from CLL patients or healthy controls, or conditioned medium of CLL cells or normal B cells. We assessed B-cell functionality for plasma cell differentiation, cellular activation, as well as proliferation and cell death.

## 2 Materials and methods

The study was approved by the ethics committee of the University Medicine Essen, vote 15-6184-BO. The participants provided their written informed consent to participate in this study.

### 2.1 Density centrifugation and CD19^+^ B-cell isolation

Peripheral blood of healthy adults (90–100 ml) (**Supplementary Table 1**) was collected in ammonium heparin monovettes (Sarstedt, Nürnbrecht, Germany). Peripheral blood mononuclear cells (PBMC) were isolated by density centrifugation. Blood was diluted with phosphate-buffered saline (PBS) + 0.5% bovine serum albumin (BSA) and layered on top of 15 ml Pancoll (Pan Biotech, Aidenbach, Germany). The samples were centrifuged for 35 min at 423 × *g* at RT (acceleration 9, deceleration 5). The PBMC were washed with PBS + 0.5% BSA and counted. The PBMC were centrifuged at 423 × *g* at 4 °C for 5 min. For B-cell isolation, the cells were incubated with anti-CD19 microbeads (Miltenyi Biotec, Bergisch Gladbach, Germany) for 15 min at 4 °C, washed with PBS, and loaded onto an equilibrated LS column (Miltenyi Biotec). The column was washed 3 times with 3 ml PBS + 0.5% BSA and eluted with 4 ml PBS + 0.5% BSA. The positive fraction was centrifuged for 5 min at 423 × *g* and 4 °C.

### 2.2 B-cell stimulation and proliferation stain

B cells were resuspended at a concentration of 1 × 10^6^ cells/ml in RPMI 1640 medium (RPMI 1640 medium, if not mentioned otherwise, contains 20% fetal bovine serum (FBS) + 1% penicillin/streptomycin (P/S)). The cells were mixed with an equal volume of eFluor670 (Thermo Fisher Scientific, Darmstadt, Germany) working solution. They were incubated for 10 min at 37 °C with 5% CO_2_ and then cooled on ice with 6–10 ml cold RPMI 1640 medium for 5 min. The cells were washed two times with 12 ml cold RPMI 1640 medium and then resuspended in either serum (see Section 2.4), conditioned medium (see Section 2.5), or normal medium as a control at a concentration of 1 × 10^6^ cells/ml. The cells were stimulated in a T-cell-dependent manner with a concentration of 10.67 µg/ml anti-Ig antibodies (IgA, IgM, IgG stimulation, Jackson Immunoresearch, Ely, UK), 5 µg/ml HA-tagged CD40L (R&D Systems, Minneapolis, MN, USA), and 5 µg/ml anti-HA antibody (R&D Systems) and incubated for 5 days at 37 °C with 5% CO_2_. The stimuli were diluted according to the manufacturer’s instructions. A fraction of the cells was set aside prior to eFluor staining for day 0 control staining.

### 2.3 Antibody staining and flow cytometry

The staining on days 0 and 5 was identical and consisted of three different stains. One sample was stained using 1 µl of 4′,6-Diamidino-2-phenylindol (DAPI) (Thermo Fisher Scientific) to stain approximately 100,000 cells in approximately 200 µl PBS + 0.5% BSA. The second sample was stained using the fluorophore-labeled antibodies anti-CD27-PECy7, anti-IgM-BV650 (for the serum assays), or alternatively anti-IgD-PerCP-Cy5.5 (for the conditioned medium assays), anti-CD80-FITC, and anti-CD86-PE antibodies. The third sample was stained using anti-CD27-PECy7 and anti-IgM-BV650 antibodies (for the serum assays), or alternatively anti-IgD-PerCP-Cy5.5 (for the conditioned medium assays), anti-CD25-FITC, and anti-CD38-APCFire750 antibodies. Antibodies used are listed in **Table 1**. The cells were incubated with the fluorophore-coupled antibodies for 15 min at 4 °C in the dark. Subsequently, the cells were washed with 4 ml PBS + 0.5% BSA and measured using a Cytoflex S flow cytometer (Beckman Coulter, Krefeld, Germany).

**Table 1:** Antibodies used in the assays.

Antibody	Clone	Fluorophore	Company	Location
Anti-CD25	M-A251	FITC	Becton Dickinson Biosciences (BD)	Heidelberg, Germany
Anti-CD86	IT2.2	PE	BD	Heidelberg, Germany
Anti-CD80	L307.4	FITC	BD	Heidelberg, Germany
Anti-CD27	M-T271	PE-Cy7	BD	Heidelberg, Germany
Anti-IgM	MHM-88	Brilliant Violet 650	BioLegend	San Diego, CA, USA
Anti-IgD	IA6-2	PerCP-Cy5.5	BioLegend	San Diego, CA, USA
Anti-CD38	HB-7	APC Fire750	BioLegend	San Diego, CA, USA
Anti-ROR1	2A2	PE	Miltenyi Biotec	Bergisch Gladbach, Germany
Anti-CD3	UCHT1	FITC	BD	Heidelberg, Germany
Anti-CD20	2H7	BV510	BioLegend	San Diego, CA, USA
Anti-CD19	HIB19	PE-Cy5	BD	Heidelberg, Germany

### 2.4 Serum collection

Serum was collected in serum-gel monovettes (Sarstedt). The monovettes were centrifuged at 423 × *g* for 15 min. The serum was sterile-filtered using a 0.2-µm filter (Sartorius, Göttingen, Germany) and frozen at − 20 °C until use in the assays. Clinical data of the patients from whom serum was used are provided in **Supplementary Table 2**.

### 2.5 Preparation of conditioned medium

Conditioned medium was prepared by incubating either CLL cells or B cells from a healthy individual in a coculture with HS-5 cells. HS-5 cells were cultivated in Dulbecco's Modified Eagle Medium (DMEM) (PAN Biotech), 10% FBS, and 1% P/S and transferred to a 24-well plate or T75-cell culture flask at a concentration of 0.3 × 10^6^ cells/ml. Each well contained 500 µl of cell suspension; for coculture with CLL cells, 10 ml of cells was added to the flask. The HS-5 cells were allowed to adhere for at least 6 h prior to adding the B cells. To obtain a 1:20 ratio of HS-5 cells to B cells, 3 × 10^6^ cells were added to the wells, and 6 × 10^7^ CLL cells were added to the flasks. The coculture was incubated for 6 days at 37 °C with 5% CO_2_. The percentage of dead cells in the supernatant, as well as of cells attached to the HS-5 cells in coculture, was determined on day 6 of coculture. DAPI was used to determine the percentage of dead cells as described above. Supernatants of the coculture were sterile-filtered using a 0.2-µm filter to remove remaining cells and stored at − 20 °C until use. To avoid a possible batch effect, we pooled the conditioned media of healthy donors 4 and 5 after sterile filtration to use the same mixture in all subsequent assays.

B cells from healthy individuals were isolated using density centrifugation and magnetic-activated cell sorting (MACS) as described above (Section 2.1). CLL cells were enriched using a receptor tyrosine kinase-like orphan receptor 1 (ROR1)-based MACS, relying on the expression of ROR1 on most CLL cells. PBMC from CLL patients were isolated using density centrifugation. After the cells were washed, counted, and centrifuged as described before, 2 µl/1 × 10^7^ cells of anti-ROR1-biotin antibody (Miltenyi Biotec) was added. The cells were incubated for 15 min at 4 °C, washed with 50 ml PBS + 0.5% BSA, and centrifuged for 5 min at 8 °C at 423 × *g*. Next, 20 µl/1 × 10^7^ cells of antibiotin microbeads (Miltenyi Biotec) were added, and washing and centrifugation after 15 min of incubation were repeated. Cells were loaded onto an equilibrated LD column (Miltenyi Biotec) and washed three times with 1 ml PBS + 0.5% BSA before eluting the CLL cells with 4 ml PBS + 0.5% BSA. In functional assays, DMEM was used instead of RPMI 1640 for the medium control, since HS-5 cells are recommended to be incubated in DMEM.

We produced conditioned medium from two healthy donors (**Supplementary Table 1**), an HS-5 only control, and three CLL patients, and used it for B cells from five healthy individuals. Clinical data of the patients from whose cells the conditioned medium was produced are provided in **Supplementary Table 2**.

### 2.6 Procedures for functional assays

We performed density centrifugation and CD19 MACS (Section 2.1) to isolate B cells. The cells (185,000–300,000 per condition) were stained using eFluor670 proliferation dye and stimulated in a T-cell-dependent manner (Section 2.2). A flow cytometric analysis of the activation markers CD80, CD86, and CD25, as well as of CD38 and DAPI to determine cell death, was performed and measured on a Cytoflex S flow cytometer on day 0 (Section 2.3) and after incubation in either 50% diluted conditioned medium (Section 2.5) or in 100% serum (Section 2.4). Proliferation was measured as the percentage of eFluor670_low_ cells. The gating strategy for cell death, proliferation, and B-cell markers is shown in **Supplementary Figures 1**, **2**.

For the titration of stimuli, half and double the amounts of stimuli as stated in Section 2.2 were used. A total of 500,000 cells were incubated for 5 days in medium (DMEM + 20% fetal calf serum (FCS) + 1% P/S) and stained using the antibodies described in Section 2.3 to determine the optimal stimulation concentrations.

### 2.7 Functional assay with cell–cell contact

Six hours prior to starting the coculture, 0.15 million HS-5 cells were seeded in a 24-well plate in 500 µl DMEM containing 20% FCS + 1% P/S. CD19^+^ B cells from the peripheral blood of two healthy donors were isolated according to **Section 2.1**. Subsequently, cells were stained with anti-CD45-APC-Cy7, anti-CD3-FITC, and anti-CD20-BV510 for 15 min at 4 °C in the dark. The cells were washed and transferred to a MACSQuant Tyto Cartridge HS (Miltenyi Biotec), and CD45^+^CD3^−^CD20^+^ cells were sorted using a MACSQuant Tyto (Miltenyi Biotec) to achieve the highest possible purity and reduce the risk of a mixed lymphocyte reaction with residual T cells when combining lymphocytes from two donors later on. CLL cells were isolated from peripheral blood starting with density centrifugation (Section 2.1), followed by enrichment of ROR1^+^ CLL cells using MACS (Miltenyi Biotec). PBMC were stained with anti-ROR1-biotin and incubated for 15 min at 4 °C in the dark. The cells were washed and incubated for another 15 min at 4 °C in the dark with antibiotin MicroBeads (Miltenyi Biotec). The cells were washed and loaded onto an equilibrated LD column (Miltenyi Biotec) and washed three times with PBS + 0.5% BSA. Subsequently, the cells were stained with anti-CD45-APC-Cy7, anti-CD3-FITC, anti-CD20-BV510, and anti-ROR1-PE, washed after incubation for 15 min, and transferred to a MACSQuant Tyto Cartridge HS to sort and purify CD45^+^CD3^−^CD20^+^ROR1^+^ CLL cells. Six million CLL cells and 3.6 million cells from healthy donors were sort-purified. The CLL cells and 3 million B cells from healthy donors were stained with eFluor450 proliferation dye to label these cells for coculture (Thermo Fisher Scientific). The remaining 0.6 million B cells from healthy donors were stained with eFluor670 proliferation dye, according to Section 2.2, to label these test cells. The 0.6 million B cells were split, and both fractions were stimulated in a TD-dependent manner according to Section 2.2. The CLL cells (3 million/500 µl in DMEM + 20% FCS + 1% P/S) and the stimulated healthy donor cells (0.3 million/300 µl in DMEM + 20% FCS + 1% P/S) were added on top of pre-seeded HS-5 cells. One milliliter of additional DMEM medium was added on top of the combined cells. The cells were incubated for 6 days at 37 °C with 5% CO_2_. On day 6, the cells were stained with fluorophore-coupled antibodies. The first stain was performed using anti-CD80-FITC, anti-CD86-PE, and anti-CD19-PE-Cy5, the second using anti-CD25-FITC and anti-CD19-PE-Cy5, and the third using anti-CD3-FITC and anti-CD19-PE-Cy5. The cells were incubated for 15 min at 4 °C in the dark, washed after, and measured on the Cytoflex S.

### 2.8 Software used and statistical evaluation

Data analysis was performed using Kaluza (Beckman Coulter) and RStudio (Posit; packages ggplot and ggpubr) software. We used a paired Wilcoxon signed-rank test for statistical analysis. Results were considered significant when *p* ≤ 0.05.

## 3 Results

### 3.1 Serum from CLL patients does not have a significant suppressive effect on differentiation, activation, proliferation, or survival of stimulated B cells from healthy individuals

B cells from healthy donors were stimulated in a T-cell-dependent fashion through B-cell receptor (BCR) crosslinking and exposure to hemagglutinin (HA)-coupled CD40L and anti-HA antibody, and exposed to sterile-filtered serum from CLL patients or healthy individuals for 5 days. Subsequently, the surface expression of CD38, CD25, CD80, and CD86 on B cells was analyzed, as well as proliferation and cell death (see **Supplementary Figure 1** for gating strategy). We selected CD80 and CD86 as activation markers with a major role as costimulatory ligands for T cells (31). They are present at low levels on resting naive B cells and are activated upon BCR and/or CD40 stimulation in T-cell-mediated immune responses (32–35). Likewise, CD25 is upregulated on activated human B cells and plays a role in inducing T-cell responses (36, 37). CD38 was selected as its high expression is a marker for plasmablasts and plasma cells. For the T-cell-dependent B-cell stimulation, we decided to use concentrations that were similarly used in earlier B-cell studies from our group (38, 39). We also tested stimulations with half or double the concentration of CD40L and antibodies. Since we observed a tendency for lower upregulation of CD80 and CD86 with lower concentrations of stimuli and higher upregulation with higher concentrations (considering both the frequencies of positive cells and the mean fluorescence intensities of positive cells), as well as proliferation of 20%–40% of cells in all conditions, we conclude that the concentration used is within a range that enables substantial up- and downregulation when studying the effects of serum or conditioned medium on B cells (**Supplementary Figure 3**).

**Figure 1:**
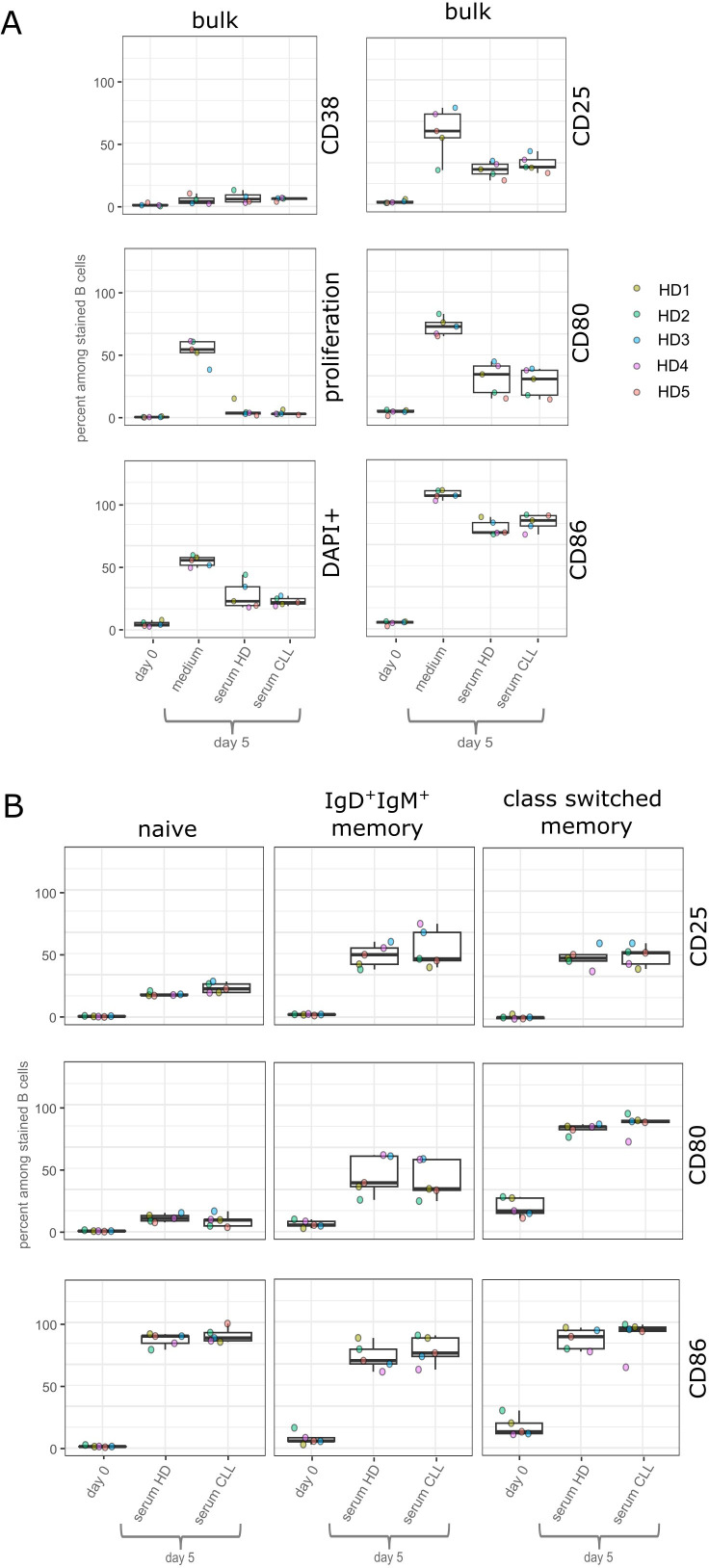
Results of assays using human serum. B cells were incubated for 5 days in serum of healthy individuals, serum of CLL patients, or RPMI 1640 medium containing 20% FBS. The percentage of cells expressing activation markers is compared with the percentage of cells expressing these markers on day 0. Statistical analysis was performed by comparing the percentages of cells that were surface-positive and incubated in serum of CLL patients versus those incubated in serum of healthy individuals using the paired Wilcoxon signed-rank test. ^*^p-value < 0.05. *n* = 5 biological replicates. Depicted is the mean value of each assay using three distinct CLL sera and three distinct sera of healthy donors (HD). **(A)** Percent of eFluor670-stained bulk B cells that express CD38, show proliferation (measured as reduced staining intensity for eFluor670), or are DAPI^+^, indicating cell death. Right, comparison of the percentage of bulk B cells that express CD25, CD80, or CD86 on the cell surface. **(B)** Percentage of eFluor670-stained B cells divided into B-cell subpopulations of naive, IgD^+^IgM^+^ memory, or class-switched memory B cells that show surface expression of CD25, CD80, or CD86.

Upon exposure of the activated B cells to serum from CLL patients or healthy donors, we did not detect a major increase in CD38 expression, which would indicate differentiation into plasmablasts or plasma cells, in any of the B-cell incubations in serum from CLL patients or healthy individuals, or in cell culture medium containing FBS used as an additional control (**Figure 1A**). The same applies to differences in proliferation of B cells or cell death in the different sera (**Figure 1A**). We did not detect proliferation of B cells in serum after 5 days of incubation, while an average of 50% of cells divided at least once in the medium control. B-cell stimulation resulted in increased frequencies of cells expressing the activation markers CD25, CD80, and CD86, as expected. Again, there was no significant difference whether B-cell cultivation was performed in serum from CLL patients or healthy donors (**Figure 1A**). The average frequencies of B cells expressing the activation markers was 29% and 25% for CD25, 25% and 28% for CD80, and 76% and 72% for CD86 for incubation with CLL serum and healthy control serum, respectively (**Figure 1A**). In the medium control, higher fractions of B cells upregulated the activation markers (**Figure 1A**).

To determine whether, within the same bulk cultivations, there are differences in the upregulation of activation markers in culture with serum from CLL patients versus healthy controls in naive or memory B cells, we also stained B cells after 5 days of cultivation for IgD and CD27, which allowed us to distinguish naive (IgM^+^IgD^+^CD27^−^), IgM^+^IgD^+^CD27^+^ memory, and class-switched memory (IgM^−^CD27^+^) B cells. This subset comparison seemed feasible because the 5-day cultivation neither induced significant CD27 upregulation nor class switching in B cells (**Supplementary Figure 4B**). A higher frequency of IgM^+^IgD^+^CD27^+^ and class-switched memory B cells than naive B cells showed upregulation of CD25 and CD80, whereas CD86 was similarly upregulated by most B cells of all three B-cell subsets (**Figure 1B**; **Supplementary Figure 5A**). One key feature of memory B cells is that these cells are more easily activated than naive B cells (40). However, even in these distinct B-cell subsets, there was no significant difference when comparing cells cultivated in CLL-derived serum or serum from healthy controls (**Figure 1B**). We additionally analyzed the geometric mean of positively gated cells for the activation markers and did not detect significant differences between cells incubated in the different sera either (**Supplementary Figure 5**).

### 3.2 Conditioned medium from CLL cells does not have a significant suppressive effect on differentiation, activation, proliferation, or survival of stimulated B cells from healthy individuals

Serum contains secreted factors and proteins from all blood cells, including but not limited to those secreted by CLL B cells. We therefore opted for further experiments using conditioned medium, containing only factors secreted by CLL cells and their feeder cell line, over an incubation period of 6 days. We used the HS-5 cell line as a stromal cell line that is widely used and known to extend CLL cell survival in coculture (26, 41). As controls, we incubated the same number of B cells from healthy donors in the same coculture system with HS-5 cells to obtain conditioned medium from normal B cells. Additionally, we used conditioned medium from only HS-5 cells to control for effects caused by factors secreted by this cell line.

We used the same experimental setup as in the serum assays, only changing the serum to 50% conditioned medium combined with 50% DMEM medium with 20% FBS (see **Supplementary Figure 2** for gating strategy). We decided to use this mixture because initial experiments with 100% conditioned medium showed a complete absence of proliferation, likely due to a lack of sufficient nutrients in the conditioned medium (data not shown).

B cells from a healthy individual were incubated in three different CLL-conditioned media (from CLL and HS-5 cells), in one conditioned medium from healthy individuals (normal B cells and HS-5 cells, pooled from two donors), in one control conditioned medium from HS-5 cells alone, and in a medium control (DMEM plus 20% FBS plus 1% P/S).

**Figure 2:**
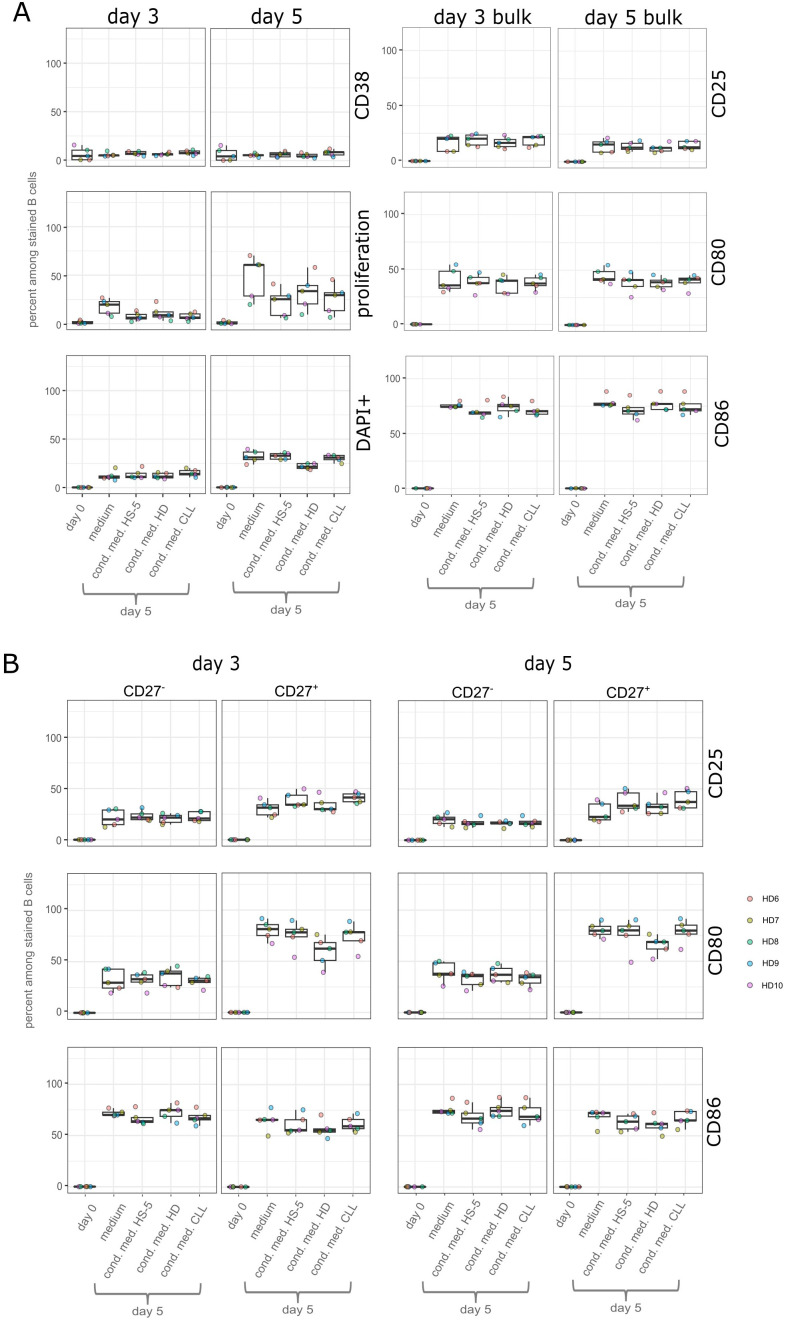
Results of assays using conditioned medium. B cells were incubated in the conditioned medium of CLL cells and HS-5 cells, in conditioned medium of B cells of a healthy donor and HS-5 cells, conditioned medium of only HS-5 cells, or in DMEM medium for 5 days. Statistical analysis was performed comparing the percentages of cells that were surface positive incubated in the three different types of conditioned media using the paired Wilcoxon signed-rank test. ^*^p-value 0.05. *n* = 5 biological replicates. Depicted is the mean value using three different CLL-conditioned media, one HS-5-only conditioned medium, and one pooled conditioned medium (of two donors) of B cells of a healthy individual. HD, healthy donor. **(A)** Percentage of eFluor-stained bulk B cells that express CD38, CD25, CD80, or CD86 on their surface. Comparison of the percentage of dead cells using DAPI or that proliferated. All markers were measured after 3 and 5 days of incubation. **(B)** Comparison of the percentage of eFluor-stained CD27^+^ or CD27^−^ B cells that express CD25, CD80, or CD86 on their surface on day 3 or 5.

We did not detect any substantial increase in CD38 expression after cell culturing for 3 or 5 days in conditioned medium (**Figure 2A**), which would indicate differentiation into plasmablasts or plasma cells. Since we did not see a difference between cells in HS-5 cell-conditioned medium, cells in conditioned medium from B cells of a healthy donor, and CLL cell-conditioned medium, we conclude that there are no inhibitory or positive effects of CLL-derived factors on normal B cells with respect to plasma cell differentiation in our setting. We also examined proliferation and cell death on days 3 and 5. There was little to no proliferation detectable on day 3, but after 5 days of incubation, we detected 25% proliferated cells in the CLL-conditioned media and 32% proliferated cells in the conditioned medium of healthy individuals, which was not significantly different (**Figure 2A**). Therefore, the amount of nutrients seemed to be sufficient for proliferation and activation of the cells, although the percentage of proliferated cells remained lower than in the medium control (48% at day 5) (**Figure 2A**). There was also no significant difference in the percentages of dead cells in any of the conditions (**Figure 2A**).

Furthermore, we examined the surface expression of the B-cell activation markers CD25, CD80, and CD86. This revealed no significant differences among the bulk B cells in HS-5 only conditions, CLL cell-conditioned medium, and conditioned medium from healthy individuals for all measured markers on day 3 as well as on day 5 (**Figure 2A**). To determine whether naive or memory B cells respond differently to stimulation and are influenced to different extents by soluble factors in the conditioned medium, we used CD27 as a marker to identify CD27^−^ (mostly) naive and CD27^+^ memory B cells (**Supplementary Figure 2**). Staining for IgM or IgD could not be used to further separate IgM^+^IgD^+^ and class-switched memory B cells, as no clear separation was observed for these markers after 5 days of cultivation. The percentages of CD27^+^ and CD27^−^ cells did not change significantly after 3 or 5 days of incubation (**Supplementary Figure 6A**). There was no significant difference in the surface expression of CD25, CD80, and CD86 detectable in CD27^+^ and CD27^−^ cells when comparing cells incubated in HS-5-only conditioned medium, CLL-conditioned medium, or conditioned medium from B cells of healthy individuals (**Figure 2B**). To analyze the fluorescent intensity of the signals, we measured the geometric mean of the positively gated cells for the activation markers and did not detect significant differences between cells incubated in the different conditioned media (**Supplementary Figure 7**). The division into CD27^−^ and CD27^+^ cells of the same dataset revealed higher, yet not significant, upregulation of CD25 and CD80 on memory B cells than on naive B cells (for CD25 only on day 3), while the percentage of CD86 showed a tendency to be higher in CD27^−^ cells on days 3 and 5 (**Supplementary Figure 6B**).

Taken together, in the assays with conditioned medium, for none of the features analyzed was there a suppressive effect of CLL-conditioned medium in comparison to conditioned medium from healthy donor B cells detectable.

Finally, we additionally performed assays incubating CLL cells with B cells from a healthy donor in direct cell–cell contact, measuring the same activation markers, cell death, and proliferation (**Supplementary Figure 8**). To avoid a mixed lymphocyte reaction, we used magnetic cell isolation and subsequently a sort-purification step to deplete T cells. There were no significant inhibitory effects on the measured markers when B cells were incubated with CLL compared with B cells from another healthy donor (**Supplementary Figure 8D**).

## 4 Discussion

### 4.1 Serum from CLL patients does not specifically impair B-cell activation or survival *in vitro*

We hypothesized that factors secreted by CLL cells could impair the functionality of the normal residual B cells and thereby contribute to the known frequent humoral immunodeficiency of CLL patients. To test this, we performed functional assays, incubating stimulated B cells from healthy donors with serum from CLL patients. We analyzed proliferation, cell death, and surface expression of key cell activation markers after 3 and 5 days. We were able to activate the cells and measure increases in surface expression of the activation markers CD25, CD80, and CD86 in cells incubated in serum. However, there was no significant difference in the upregulation of activation markers when comparing B-cell cultivation performed in serum from CLL patients or healthy donors. There was also no increased cell death of B cells cultivated in serum from CLL patients. We did not detect proliferation of B cells when we incubated cells in serum, whereas B cells proliferated in the presence of cell culture medium and FBS. Possibly, medium plus FBS is a richer source of nutrients for B cells *in vitro* than human serum. Importantly, however, there was no significant difference in the behavior of B cells when comparing serum from CLL patients and healthy controls.

### 4.2 Conditioned medium from CLL cells does not specifically inhibit activation or proliferation of normal B cells or induce their death

To further validate our observations and test whether factors specifically secreted from CLL cells have an inhibitory influence on normal B cells *in vitro*, we performed a second set of experiments in which we compared the effects of conditioned medium from CLL cells and healthy donor B cells on B cells from healthy individuals. For these experiments, the CLL cells (and consequently also the normal B cells) had to be cultivated on stromal cells to maintain viability for the production of conditioned medium. Therefore, the effect of the stromal cells had to be controlled, as they produce soluble factors (26). To control for the effects of HS-5-secreted factors, we also incubated normal B cells in conditioned medium from HS-5 cells, in addition to a control with cell culture medium and FBS instead of conditioned medium. In these various cultures, the B cells showed upregulation of activation markers and proliferation, but there was no inhibitory effect of CLL-derived conditioned medium in comparison with the conditioned medium from HS-5 cells or HS-5 cells plus normal B cells. There was also no increased cell death in the cultures with CLL-derived conditioned medium. We conclude that also soluble factors secreted by CLL cells *in vitro* do not have an inhibitory effect on normal B cells, at least for the variables we analyzed. Our results are further strengthened by additional assays in which B cells from a healthy donor were incubated in direct cell–cell contact with CLL cells, which also showed no inhibitory effects of the presence of CLL cells.

### 4.3 Limitations of the study

We included three distinct sera and three distinct conditioned media per assay, but were limited by the amount of cells isolated from 100 ml of blood. Nevertheless, our assays showed reproducible results that were comparable among the different conditioned media from healthy individuals. The data furthermore did not show major variability in cells incubated in the different CLL sera or conditioned medium. Adding more patients to our cohort would further strengthen our results. Due to the limited amount of B cells, we were restricted to two time points of measurement. Consequently, we cannot exclude that soluble CLL-derived factors could influence B-cell function at other time points. Additionally, our serum assays did not show proliferation or plasma cell differentiation in the healthy control, so that for these features, a potential inhibitory effect of CLL serum was *a priori* not detectable. Using additional stimulating factors (e.g., IL-21 or BAFF) for B-cell stimulation might address this limitation, but even stronger stimulation could also mask the inhibitory effects of CLL-secreted factors. We cannot draw final conclusions on the effect of CLL cells on the normal residual B-cell compartment *in vivo* due to limited sample size and the assessment of only selected readouts, including activation markers, proliferation, and cell death. There is the possibility that neither cell–cell contact between CLL cells and normal residual B cells nor secreted factors influences B cells directly, but that they are rather influenced indirectly by other immune cells, for example, T cells, which have a known dysfunction in CLL.

### 4.4 Conclusions

In conclusion, we performed assays using CLL patients’ sera or conditioned medium from purified CLL cells to test whether and to what extent CLL-derived soluble factors directly influence B cells. The analysis suggests no major direct inhibitory effects of CLL-derived soluble factors on normal mature B cells. Further studies are needed to understand whether and how normal B cells are influenced in CLL patients and what causes the humoral dysfunction in CLL patients. 

## Data Availability

The original contributions presented in the study are included in the article/**Supplementary Material**. Further inquiries can be directed to the corresponding author.
