# Supplementary material for: CLL cell-derived soluble factors do not influence the functionality of normal B cells
Source: Front Immunol. 2026 May 15;17:1794418. doi: 10.3389/fimmu.2026.1794418 (PMC13219295; doi:10.3389/fimmu.2026.1794418)
Supplement: Supplementary file 1 [file DataSheet1.pdf]

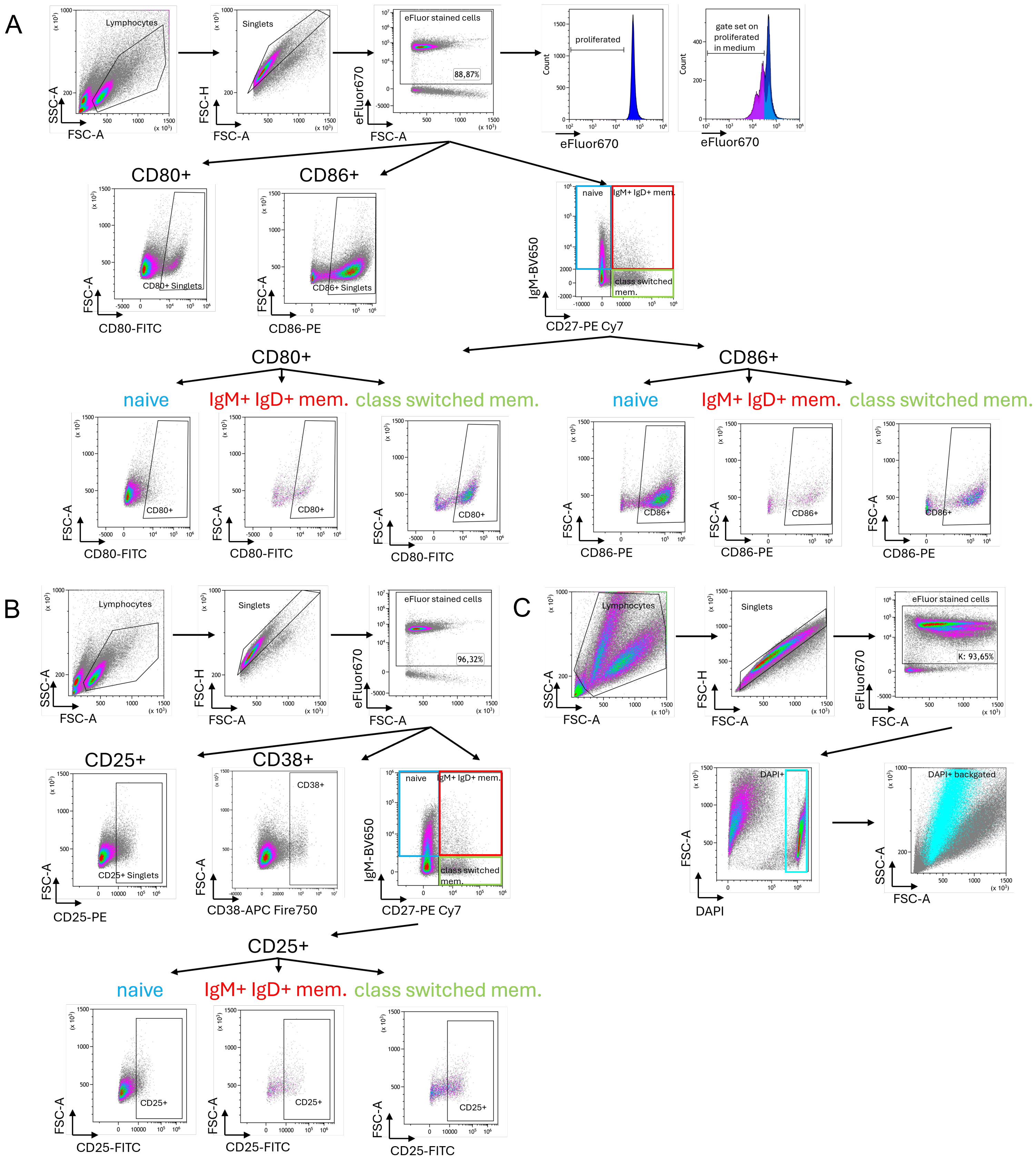

Suppl. Fig. 1: **Gating strategy of the assays using human serum**. Example of cells incubated in serum of healthy donors on day 5 of incubation

A) Gating strategy of the proliferation and of CD80$^+$ and CD86$^+$ B cells.

B) Gating strategy of CD25$^+$ and CD38$^+$ B cells

C) Gating strategy of DAPI$^+$ B cells, marking dead cells.
