# Supplementary figures and images for: CLL cell-derived soluble factors do not influence the functionality of normal B cells
Source: Front Immunol. 2026 May 15;17:1794418. doi: 10.3389/fimmu.2026.1794418 (PMC13219295; doi:10.3389/fimmu.2026.1794418)

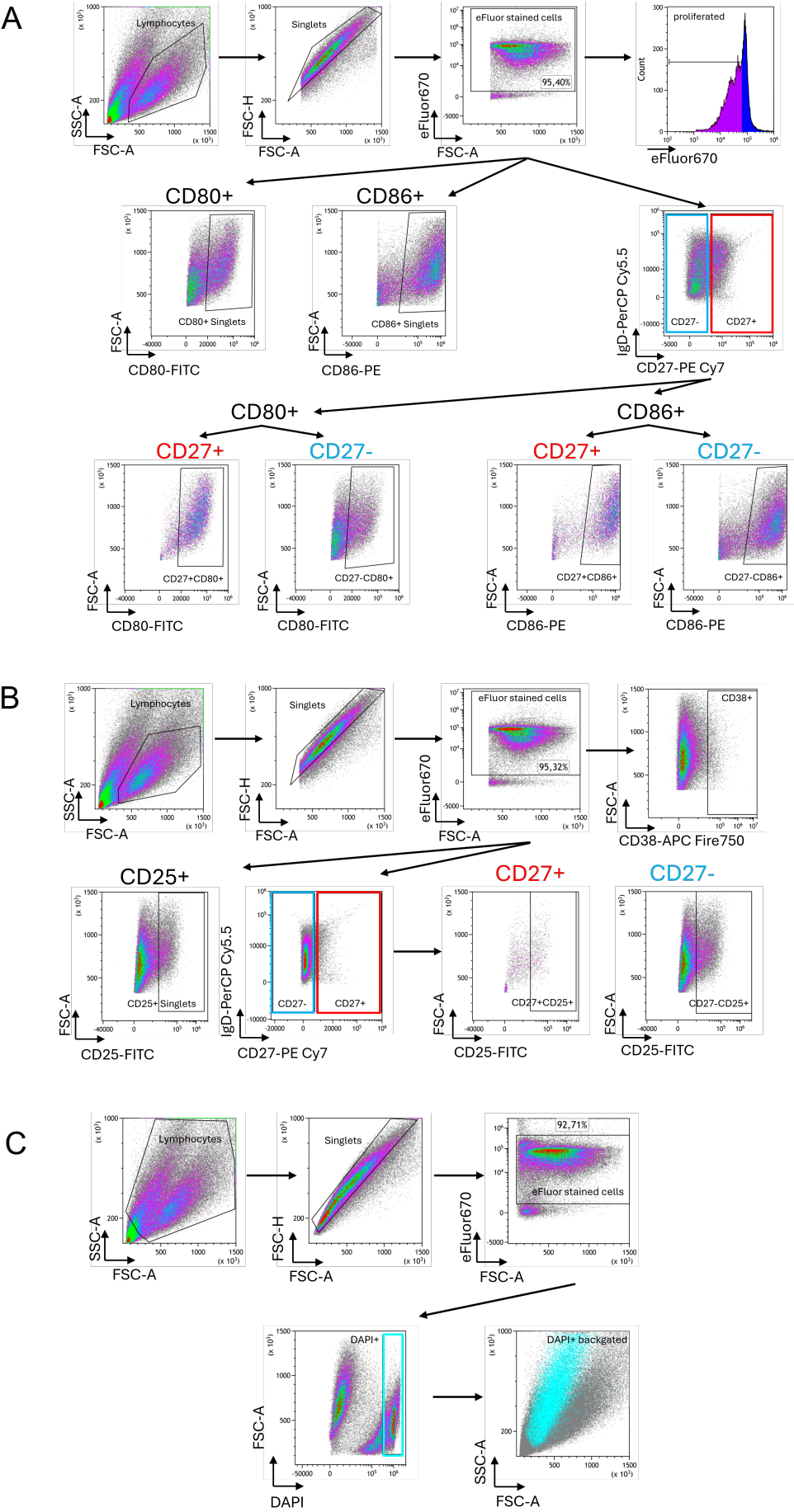

Supplement: Supplementary file 2 [file DataSheet2.pdf]

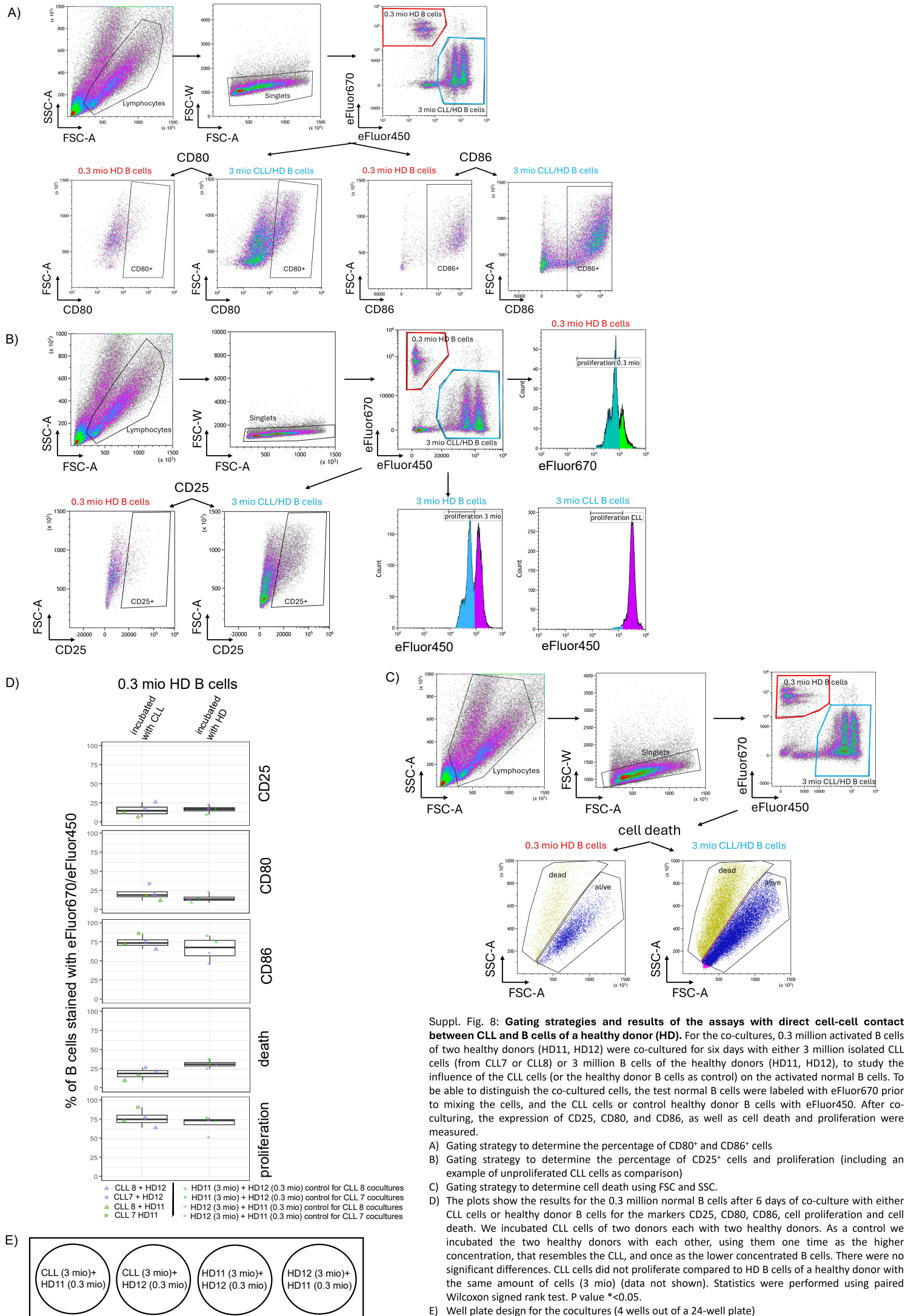

Supplement: Supplementary file 8 [file DataSheet8.pdf]
