# Supplementary material for: CLL cell-derived soluble factors do not influence the functionality of normal B cells
Source: Front Immunol. 2026 May 15;17:1794418. doi: 10.3389/fimmu.2026.1794418 (PMC13219295; doi:10.3389/fimmu.2026.1794418)
Supplement: Supplementary file 3 [file DataSheet3.pdf]

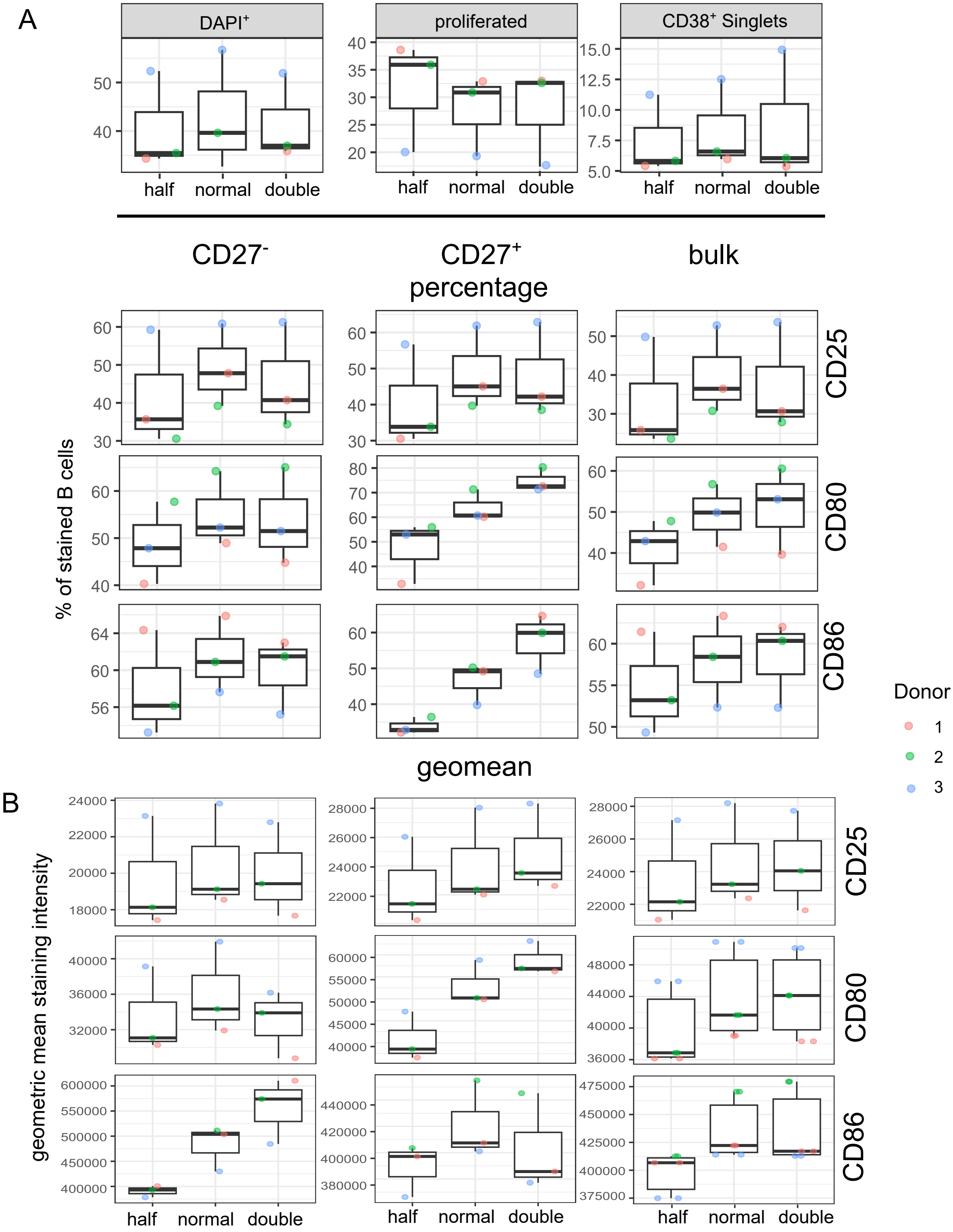

Suppl. Fig. 3: **Titration experiments of the stimulation reagents used to test the optimal concentration to perform the assays with.** We tested half the amount of stimulation (5.34 µg/ml anti-Ig antibody, 2.5 µg/ml HA-tagged CD40L and 2.5 µg/ml anti-HA antibody), the normal amount stated in the methods section (10.67 µg/ml anti-Ig antibody, 5 µg/ml HA-tagged CD40L and 5 µg/ml anti-HA antibody) and double the amount (21.34 µg/ml anti-Ig antibody, 10 µg/ml HA-tagged CD40L and 10 µg/ml anti-HA antibody). Analysed were the percentage (A) or geometric mean of the positively set gate (B) of CD25, CD80 and CD86 expressing cells, as well as the percentage of those expressing CD38, the DAPI+ cells and the eFluor670 low proliferated cells. Statistical analysis was performed using paired Wilcoxon signed rank test, P value *<0.05
