# Supplementary material for: CLL cell-derived soluble factors do not influence the functionality of normal B cells
Source: Front Immunol. 2026 May 15;17:1794418. doi: 10.3389/fimmu.2026.1794418 (PMC13219295; doi:10.3389/fimmu.2026.1794418)
Supplement: Supplementary file 4 [file DataSheet4.pdf]

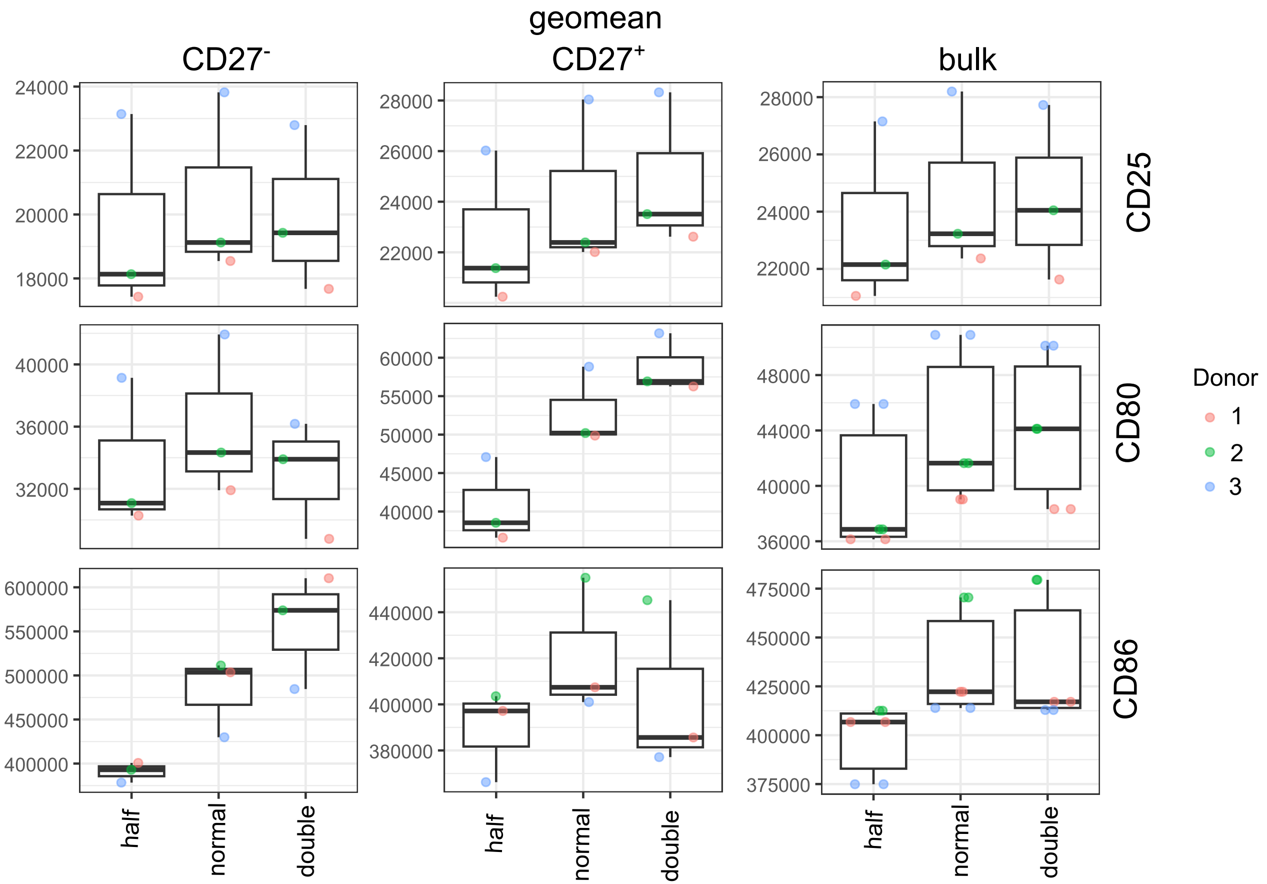

Suppl. Fig. 4: **Titration experiments of the stimulation reagents used to test the optimal concentration to perform the assays with**. We performed assays using half the amount of anti-Ig, CD40L-HA and anti-HA, the normal amount stated in the methods section and double the amount. Analysed was the geometric mean of CD25, CD80 and CD86 expressing cells in the $CD27^{+}$, the $CD27^{-}$ and in bulk B cells. Statistical analysis was performed using paired Wilcoxon signed rank test, P value *<0.05
