# Supplementary material for: CLL cell-derived soluble factors do not influence the functionality of normal B cells
Source: Front Immunol. 2026 May 15;17:1794418. doi: 10.3389/fimmu.2026.1794418 (PMC13219295; doi:10.3389/fimmu.2026.1794418)
Supplement: Supplementary file 5 [file DataSheet5.pdf]

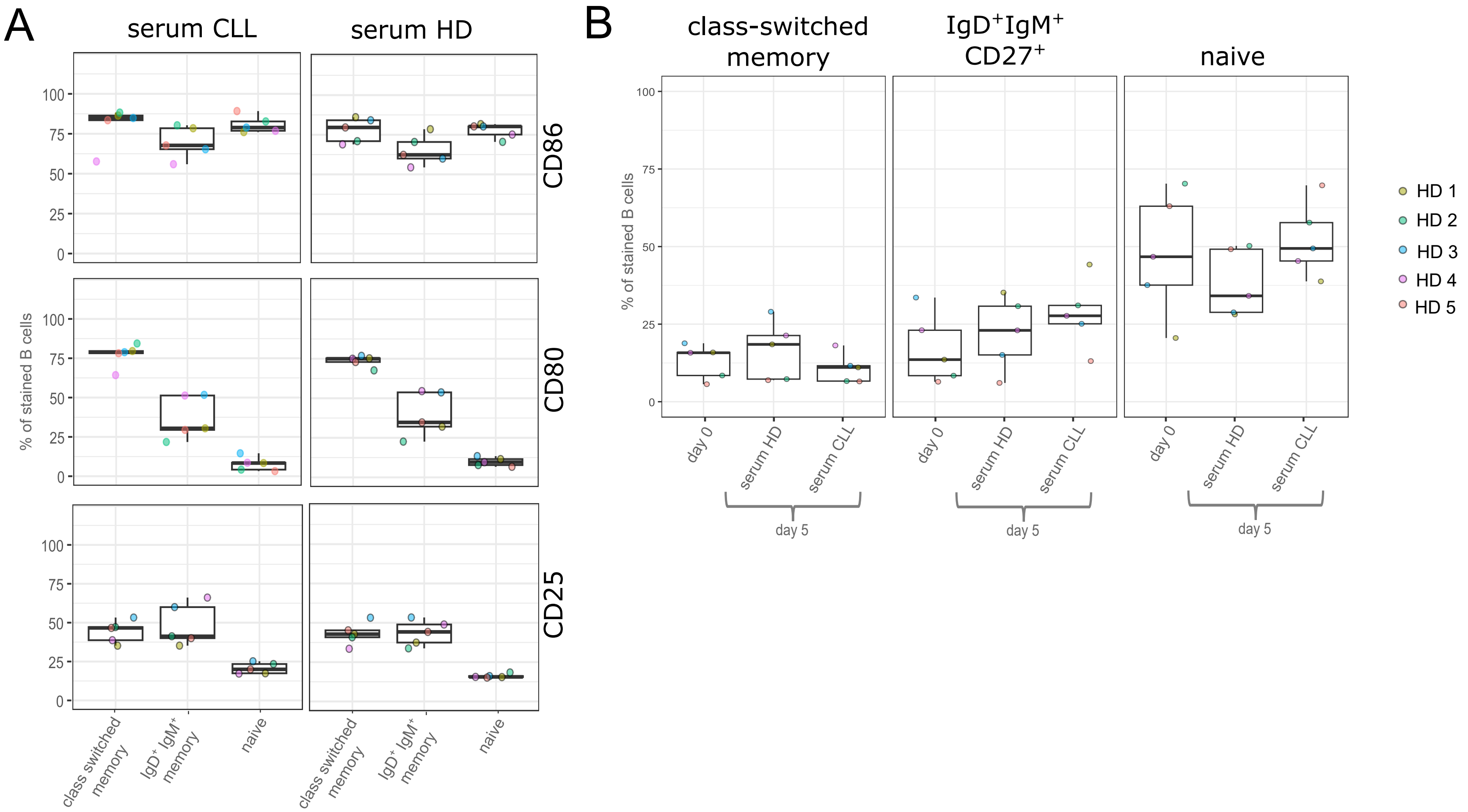

Suppl. Fig. 5: **Additional comparisons that were analysed using the same data set of the assays using human serum (Fig. 1).** n=5 biological replicates. Depicted is the mean value of each assay using three distinct CLL and three distinct sera of healthy donors. HD = healthy donor. Statistical analysis was performed using paired Wilcoxon signed rank test. P value *<0.05**.**

A) percentage of B cells that express the activation markers CD25, CD80 and CD86 compared between the different B-cell subpopulations of naïve and memory B cells on day 5.

B) percentage of B-cell subpopulations (naïve, $IgD^+IgM^+CD27^+$ memory B cells and $CD27^+$ class-switched memory B cells) on day 0 and 5 days of incubation in serum.
