# Supplementary material for: CLL cell-derived soluble factors do not influence the functionality of normal B cells
Source: Front Immunol. 2026 May 15;17:1794418. doi: 10.3389/fimmu.2026.1794418 (PMC13219295; doi:10.3389/fimmu.2026.1794418)
Supplement: Supplementary file 6 [file DataSheet6.pdf]

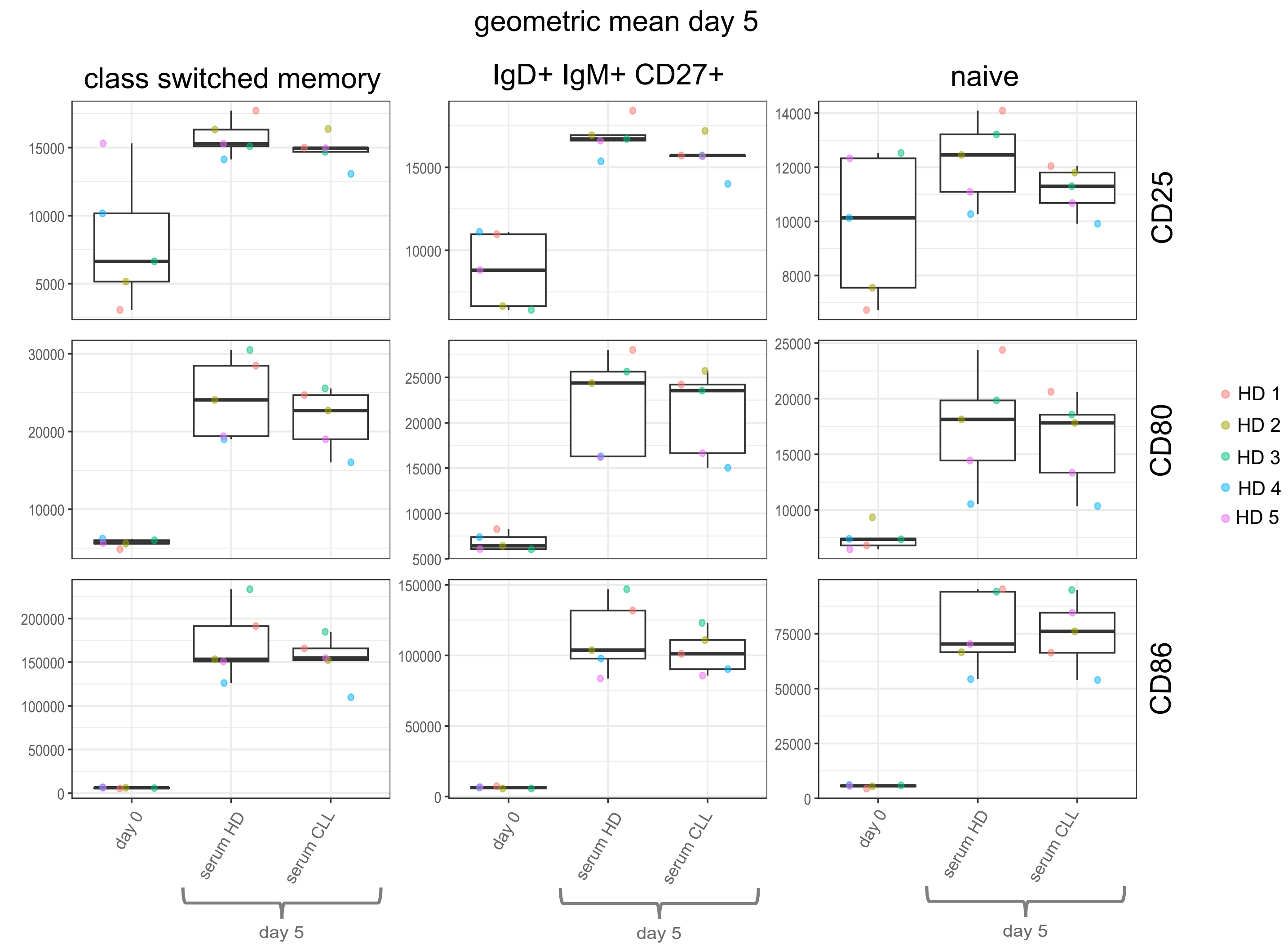

Suppl. Fig. 6: **Additional comparisons that were analyzed using the same gating of the assays using human serum but analyzing the geometrical mean of the positve fraction of cells (Fig. 1).**
n=5 biological replicates. Depicted is the mean value of each assay using three distinct CLL and three distinct sera of healthy donors. HD = healthy donor. Statistical analysis was performed using paired Wilcoxon signed rank test. P value *<0.05**.**

Geomean of B cells that express the activation markers CD25, CD80 and CD86 compared between the different B-cell subpopulations of naïve and memory B cells.
