# Supplementary material for: CLL cell-derived soluble factors do not influence the functionality of normal B cells
Source: Front Immunol. 2026 May 15;17:1794418. doi: 10.3389/fimmu.2026.1794418 (PMC13219295; doi:10.3389/fimmu.2026.1794418)
Supplement: Supplementary file 7 [file DataSheet7.pdf]

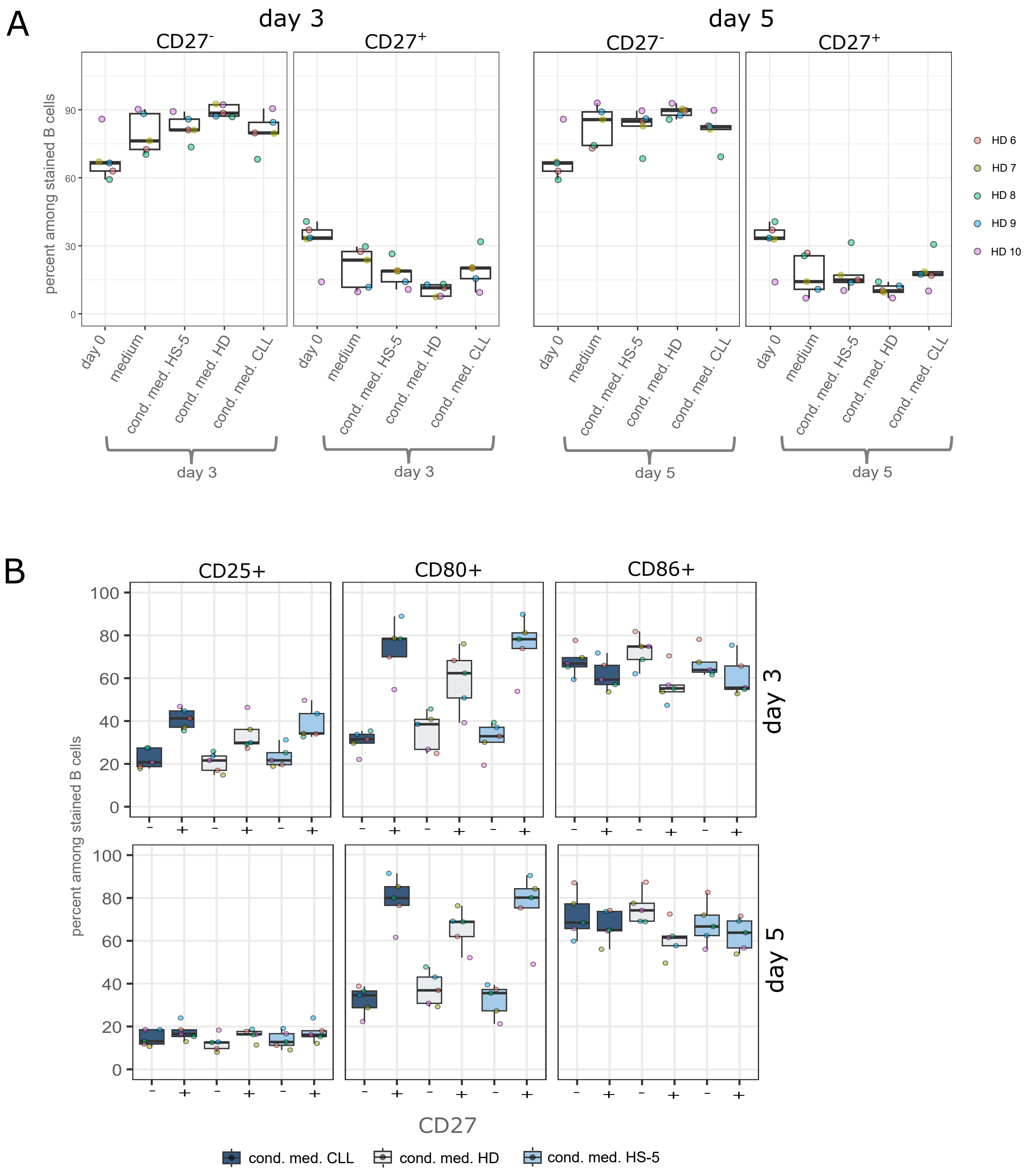

Suppl. Fig. 7: **Additional comparisons that were analyzed using the same data set of the assays using conditioned medium (Fig. 2).** Statistical analysis was performed using paired Wilcoxon signed rank test, P value *<0.05. n=5 biological replicates, depicted is the mean value of using three different CLL and three different conditioned media, one HS-5 only conditioned medium and one pooled conditioned medium of B cells of a healthy individual. HD = healthy donor.

A) Percentage of B cells that express the activation markers CD25, CD80 or CD86 in the groups of CD27⁻ and CD27⁺ B cells on day 3 and on day 5.

B) Comparison of the percentages of CD25, CD80 or CD86 expressing B cells in the different conditioned media between CD27⁻ and CD27⁺ cells on day 3 and on day 5.
