# Supplementary material for: CLL cell-derived soluble factors do not influence the functionality of normal B cells
Source: Front Immunol. 2026 May 15;17:1794418. doi: 10.3389/fimmu.2026.1794418 (PMC13219295; doi:10.3389/fimmu.2026.1794418)
Supplement: Supplementary file 9 [file DataSheet9.pdf]

participant healthy individual	sex	age	assay
HD1	W	26	serum
HD2	W	26	serum
HD3 (same donor as HD8 and HD11)	W	53	serum
HD4 (same donor as HD6)	M	43	serum
HD5 (same donor as HD11)	M	50	serum
HD6 (same donor as HD4)	M	43	conditioned medium
HD7	W	26	conditioned medium
HD8 (same donor as HD3 and HD11)	W	53	conditioned medium
HD9	W	42	conditioned medium
HD10	M	36	conditioned medium
HD11 (same donor as HD5)	M	50	cell-cell-contact
HD12 (same donor as HD 3 and 8)	W	53	cell-cell-contact
			**production of conditioned medium**
HD13 (same as donor 7)	W	26	
HD14 (same as Donor 2)	W	26	pooled for the diluted assays

Suppl. Table 1: Healthy donor participant information. Cells were used either to perform assays using human serum/conditioned medium or for the production of conditioned medium
