# Supplementary material for: CLL cell-derived soluble factors do not influence the functionality of normal B cells
Source: Front Immunol. 2026 May 15;17:1794418. doi: 10.3389/fimmu.2026.1794418 (PMC13219295; doi:10.3389/fimmu.2026.1794418)
Supplement: Supplementary file 10 [file DataSheet10.pdf]

CLL	experiment	disease stage	IGHV status	genetic aberrations	time since first diagnosis [months]	hypogamma-globulinemia	leukocyte count /µl	lymphocyte count /µl	treatment
1	serum	Binet A	mutated	no del17p /TP53 Mut	41	no	40290	17300	no
2	serum	Binet A	n/a	del13q14	166	yes	29750	24980	treatment in 2010 and 2016
3	serum	Binet C	mutated	no del17p /TP53 Mut	82	no	198080	101000	no
4	cond. medium	Binet C	mutated	no del17p /TP53 Mut	12	no	32490	30360	no
5	cond. medium	Binet A	mutated	none	172	no	53500	50590	no
6	cond. medium	Binet A	mutated	no del17p /TP53 Mut	47	no	48430	17000	no
7	cell-cell-contact	Binet C	unmutated	trisomy 12	30	no	25600	16500	no
8	cell-cell-contact	Binet A	mutated	none	5	no	17900	12530	no

Suppl. Table 2: Clinical data of the CLL patients whose serum or cells were used to generate conditioned medium or for the assays. Hypogammaglobulinemia is present if IgG<6.5 g/l. If only del17p/TP53 was covered by diagnostics and no further genetic aberrations were analysed „no del17p/TP53 Mut“ was added to the column. The lymphocyte count can be inaccurate due to smudge CLL cells in the blood smear.
